# Time pressure predicts decisional regret in men with localized prostate cancer: data from a longitudinal multicenter study

**DOI:** 10.1007/s00345-021-03727-0

**Published:** 2021-05-22

**Authors:** Caren Hilger, Martin Schostak, Isabella Otto, Friederike Kendel

**Affiliations:** 1 Gender in Medicine (GiM), Charité – Universitätsmedizin Berlin, corporate member of Freie Universität Berlin and Humboldt-Universität zu Berlin, Seestraße 73, 13347 Berlin, Germany; 2 Urooncology, Robotic-Assisted and Focal Therapy, Clinic of Urology, University Clinic, Otto-von-Guericke-University Magdeburg, Leipziger Straße 44, 39120 Magdeburg, Germany

**Keywords:** Decisional regret, Erectile functioning, Localized prostate cancer, Satisfaction with sexual life, Time pressure

## Abstract

**Purpose:**

A substantial proportion of men with localized prostate cancer (lPCa) later regret their treatment decision. We aimed to identify factors contributing to decisional regret.

**Methods:**

We conducted a longitudinal study, in which men with lPCa were surveyed at four measurement points: T0 (baseline) = prior to treatment; T1 = 6; T2 = 12; T3 = 18 months after baseline. *χ*^2^-tests and independent *t*-tests were used to compare men undergoing different treatments [Active Surveillance (AS) vs. local treatment]. Logistic regression models were fitted to investigate the associations between predictors (*time pressure, information provided by the urologist, impairment of erectile functioning, satisfaction with sexual life*) and the criterion *decisional regret*.

**Results:**

At baseline, the sample included *N* = 176 men (AS: *n* = 100; local treatment: *n* = 76). At T2 and T3, men after local therapies reported higher regret than men under AS. Decisional regret at T3 was predicted by time pressure at baseline (OR 2.28; CI 1.04–4.99; *p* < 0.05), erectile dysfunction at T2 and T3 (OR 3.40; CI 1.56–7.42; *p* < 0.01), and satisfaction with sexual life at T1–T3 (OR 0.44; CI 0.20–0.96; *p* < 0.05).

**Conclusions:**

Time pressure, erectile dysfunction, and satisfaction with sexual life predict decisional regret in men with lPCa. Mitigating time pressure and realistic expectations concerning treatment side effects may help to prevent decisional regret in PCa survivors.

**Trial registration number:**

DRKS00009510; date of registration: 2015/10/28.

**Supplementary Information:**

The online version contains supplementary material available at 10.1007/s00345-021-03727-0.

## Introduction

With a global incidence of about 1.3 million cases in 2018, prostate cancer (PCa) is the second most common cancer type in men [1]. More than half of the diagnosed prostate carcinomas are localized with a very good prognosis [2]. Various treatment strategies with curative intent are recommended for localized prostate cancer (lPCa) of low and intermediate risk: radical prostatectomy (RP), radiotherapy (RT) and active surveillance (AS). These strategies do not differ in mortality [3], but do differ significantly in side effects [4]. In RP (i.e. surgical removal of the prostate), common side effects are erectile dysfunction and incontinence. Possible side effects of RT encompass erectile dysfunction, urinary and rectal problems. Over time, the side effects decrease, although baseline levels are often not attained [5]. In AS, local treatment is often delayed until a predefined level of disease progression occurs. In AS, psychological side effects, such as anxiety, may occur [4, 6].

Up to one-third of patients with PCa later regret their initial treatment decision [7, 8]. Several studies with PCa patients have shown that erectile dysfunction after treatment is predictive of later decisional regret [9, 10]. In these studies, erectile dysfunction was assessed with questionnaires focusing on physical sexual functioning (e.g. erection firmness). However, this functional aspect is only one facet of sexuality and does not necessarily reflect how satisfied men are with their sexuality. Although satisfaction with sexual life is likely to play a relevant role in subjective quality of life, research on this topic has been scarce. In addition, an informed treatment decision has been shown to be associated with less regret [11]. As the urologist is still the most important information source [12], the information provided by the urologist may be particularly important for decisional regret.

Informed decision-making that carefully weighs all pros and cons of a treatment strategy takes time [11, 13]. Experimental studies in other contexts have shown that time pressure is associated with less adherence to guidelines [14] and greater decisional regret [15]. Although lPCa often does not require immediate action, the word “cancer” alone may create a desire for rapid local treatment in many patients [16]. Given the resulting time pressure, it is a challenge to carefully discuss the respective side effects of all treatment options. This is volatile because many side effects can permanently affect quality of life.

Surprisingly, the impact of time pressure on decisional regret has not yet been studied in the context of PCa. In addition, most studies concentrated on functional aspects of sexuality. The present study aimed to analyze the association of psychological variables with decisional regret in men with lPCa. We assumed that higher time pressure, less information by the urologist, higher impairment of erectile functioning and lower satisfaction with sexual life were associated with higher levels of regret. As erectile functioning is more frequently impaired after local treatments for lPCa, we further assumed that men after local treatment experience more regret than men under AS.

## Methods

### Study design and participants

In this prospective, non-interventional, multicentre study (33 urology clinics and practices), men who were ≤ 80-years old and diagnosed with low-/intermediate-risk PCa (TNM: ≤ T2a; PSA: ≤ 10 ng/ml; ISUP Grade ≤ 2) within the last six months were eligible to participate. Data were collected using self-report questionnaires at four measurement points: T0 (baseline) = prior to treatment initiation; T1 = six; T2 = twelve; T3 = 18 months after baseline. Follow-up questionnaires were mailed to participants. Clinical data at baseline (tumor category, ISUP Grade, PSA value, date of diagnosis, comorbidities) were provided by clinicians. Over the course of the study, no further clinical data were collected, such as how the AS strategy was implemented in detail (MRI, re-biopsies). All participants agreed in a written form to take part before enrollment. Ethical approval for this study was obtained from the Charité—Universitätsmedizin Berlin (EA1/242/13).

### Materials and main outcome measures

*Decisional regret* was assessed at T1–T3 using the Decision Regret Scale [17]. A score ranging from 0 to 100 can be calculated with higher values indicating higher regret. There is no validated cutoff value for clinically relevant regret. Due to a right-skewed distribution, we dichotomized the score (0–5: no regret vs. ≥ 6: at least some amount of regret) *Impairment of erectile functioning* was measured with one item adapted from Johannson et al. [18] and *satisfaction with sexual life* with one item adapted from van den Bergh et al. [19]*.* Perceived *time pressure* was assessed at baseline using a self-constructed scale consisting of four items (e.g. *“When choosing my treatment strategy, I took as much time as I needed”*). Subjective *information by the urologist* was captured with a self-constructed single item (“*Do you feel that your urologist has provided you with sufficient information?*”). For each variable, higher values indicate a higher level in the respective characteristic.

### Statistical analysis

Men undergoing local treatment (RP, RT) were compared with men under AS regarding sociodemographic and clinical characteristics using *χ*^2^-tests and independent *t*-tests/Mann–Whitney *U* tests. To investigate changes in satisfaction with sexual life over time we conducted ANOVA with repeated measures. To analyze how impairment of erectile functioning and decisional regret develop over time, we computed *McNemar* tests. Logistic regression models were fitted to investigate the associations between predictor variables (*time pressure, information by the urologist, impairment of erectile functioning, satisfaction with sexual life*) and *decisional regret*. In a first step, control variables (age, partner status, ISUP Grade, TNM category) and in a second step, the respective predictor were entered into the model. Nominal and ordinal scaled variables were dichotomized. Control and predictor variables were pre-tested for multicollinearity. All analyses were conducted with IBM SPSS Statistics (Version 25). An alpha level of *p* < 0.05 indicated statistical significance for all analyses.

## Results

At baseline, the final sample comprised *N* = 176 men (AS: *n* = 100; local treatment: *n* = 76). Over time, 10.1% of participants dropped out (T1: *N* = 167; T2: *N* = 164; T3: *N* = 160; Figure A1). Sample characteristics and study variables over time are depicted in Table 1. Treatment groups did not differ regarding sociodemographic characteristics and clinical variables, except for ISUP Grade (*p* < 0.001, *Cramér’s V* = 0.42).

**Table 1:** Sample characteristics at baseline (T0) and study variables according to treatment group over time (T0–T3)

	Total (*n* = 176)	AS (*n* = 100)	Local (*n* = 76)	*p*
Sociodemographic data				
Age, years, *M *(SD)	65.5 (7.4)	66.2 (7.0)	65.0 (7.7)	0.13
Living with partner, *n* (%)	154 (88.0)	90 (90.9))	64 (84.2)	0.18
Higher education, *n* (%)	93 (53.8)	56 (56.6)	37 (50.0)	0.39
Still working vs. retired, *n* (%)	58 (33.1)	29 (29.3)	29 (38.2)	0.22
Time since treatment decision (weeks), *M *(SD)	9.0 (8.6)	9.1 (8.9)	9.0 (8.3)	0.96
Risk classification				
ISUP Grade (low = 1), *n* (%)	135 (77.1)	91 (91.0)	44 (58.7)	< 0.001
PSA, *M *(SD)	5.9 (2.6)	5.9 (2.3)	5.9 (3.0)	0.95
TNM, (T1a–c), *n* (%)	163 (93.1)	94 (94.0)	69 (92.0)	0.60
Number of comorbidities, *M *(SD)	0.3 (0.9)	0.2 (0.5)	0.4 (1.2)	0.26
Time pressure, *M *(SD)	2.0 (0.6)	1.9 (0.5)	2.0 (0.6)	0.08
Moderate to high, *n *(%)	48 (31.4)	22 (25.6)	26 (38.8)	
Information provided by urologist, * Mdn *(*IQR*)	4 (1)	4 (1)	4 (1)	0.20
Rather to completely sufficient, *n *(%)	161 (91.5)	90 (90)	71 (93.4)	
Impairment of erectile functioning				
T0				
* Mdn (IQR)*	2 (1)	2 (1)	2 (2)	0.96
Moderate to severe, *n *(%)	39 (23.6)	22 (22.7)	17 (25)	
T1				
* Mdn (IQR)*	2 (2)	2 (2)	3 (2)	< 0.001
Moderate to severe, *n *(%)	78 (47.9)	21 (26.3)	57 (68.7)	
T2				
* Mdn (IQR)*	3 (2)	2 (2)	3 (2)	< 0.001
Moderate to severe, *n *(%)	83 (52.2)	20 (30.8)	63 (67.0)	
T3				
* Mdn (IQR)*	3 (2)	2 (1)	3 (2)	< 0.001
Moderate to severe, *n *(%)	96 (60.8)	25 (43.1)	71 (71.0)	
Satisfaction with sexual life				
T0				
* Mdn (IQR)*	4 (1)	3 (1)	4 (1)	0.69
Moderate to high, *n *(%)	137 (80.1)	81 (81.8)	56 (77.8)	
T1				
* Mdn (IQR)*	3 (2)	3 (2)	3 (2)	0.01
Moderate to high, *n *(%)	104 (63.0)	58 (71.6)	46 (54.8)	
T2				
* Mdn (IQR)*	3 (2)	4 (2)	3 (2)	0.02
Moderate to high, *n *(%)	104 (65.4)	48 (72.7)	56 (60.2)	
T3				
* dn (IQR)*	3 (2)	3 (2)	3 (2)	0.91
Moderate to high, *n *(%)	96 (62.3)	38 (66.7)	58 (59.8)	
Decisional regret (0–100)				
T1				
* M *(SD)	12.5 (17.5)	9.0 (12.2)	14.1 (19.4)	0.27
Mild to severe, *n *(%)	51 (47.7)	14 (41.2)	37 (50.7)	
T2				
* M *(SD)	9.6 (14.4)	5.6 (11.5)	11.6 (15.3)	0.001
Mild to severe, *n *(%)	52 (40.3)	10 (23.8)	42 (48.3)	
T3				
* M *(SD)	11.7 (14.0)	8.3 (11.9)	13.5 (14.7)	0.03
Mild to severe, *n *(%)	62 (47.0)	15 (31.9)	47 (55.3)	

T0: *N* = 176, AS, *n* = 100, local, *n* = 76; T1: *N* = 167, AS, *n* = 81, local, *n* = 86; T2: *N* = 164, AS, *n* = 68, local, *n* = 96; T3: *N* = 160, AS, *n* = 58, local, *n* = 102; Time pressure, range: 1–4; Information provided by urologist, range: 1–4; Impairment of erectile functioning, range: 1–4; Satisfaction with sexual life, range: 1–5; Decisional regret, range: 0–100

*AS* surveillance, *RP* radical prostatectomy, *RT* radiation therapy, *M *arithmetic mean, *SD *standard deviation, *Mdn *median, *IQR*  interquartile range

At T0, men under AS did not differ from men undergoing local treatment in erectile functioning (*p* = 0.96). After local treatment, however, men reported higher impairment compared to men undergoing AS (Table 1). Subgroup analyses showed that men after RP reported the strongest impairment compared to RT and AS (Table A2). Impairment of erectile functioning was lower at T0 compared to all follow-up time points (*p* < 0.001). Impairment of erectile functioning at 18 months was higher compared with T1 (*p* = 0.001) and T2 (*p* = 0.04).

Before treatment (T0) and 18 months later (T3) the two treatment groups did not differ in satisfaction with sexual life. At T1 (*p* = 0.01) and T2 (*p* = 0.02), however, men under local treatment were less satisfied than men under AS (Table 1). Men after RP reported being least satisfied at these measurement times compared with men under AS and RT (Table A2). Satisfaction varied over time (*p* < 0.001, *f* = 0.28): men reported higher satisfaction at baseline compared to all follow-up measurements (*p* = 0.001–0.005).

Almost half of participants (40.3–47.7%) reported at least some amount of decisional regret (Table 1). At T2 and T3, men after local therapies reported higher decisional regret than men under AS (Table 1). Men after RP regretted their treatment decision more than men under AS (Table A2).

After controlling for sociodemographic and clinical parameters, time pressure at T0 predicted decisional regret at T3 (*p* = 0.039), a trend was found for T2 (*p* = 0.06). Information by the urologist at T0 did not predict regret. Impairment of erectile functioning at T2 (*p* = 0.036) and T3 (*p* = 0.002) predicted regret at T3. All associations between satisfaction with sexual life and regret were significant, except for satisfaction at T1 predicting regret at T2 (Table A1).

## Discussion

The aim of our study was to identify factors explaining decisional regret in men with lPCa. To our knowledge, this is the first study highlighting the role of time pressure in this context. Our key finding is that time pressure at the time of diagnosis, in addition to impairment of erectile functioning and satisfaction with sexual life, is predictive of later decisional regret. Comparing the three treatment strategies RP, RT, and AS, we found that decisional regret was most pronounced in men after RP.

Our results show that almost half of men with lPCa subsequently regret their treatment decision at least to some amount. This is a higher proportion than in previous studies with between four [7] and 31% [8] of men reporting decisional regret. These large differences could be due to different cut-offs on the Decision Regret Scale. There is no consensus yet which cut-off value for the Decision Regret Scale [17], which ranges from 0 to 100, is considered clinically relevant. While van Stam et al. [20] set a cut-off value at 30, Wilding et al. [9] recommend a more refined classification, distinguishing between milder and stronger regret. In their study, two thirds of men reported at least a mild expression of decisional regret. We also chose a conservative cut-off that includes mild levels of regret. This approach takes into account that many men have difficulties expressing their feelings openly [21]. Another reason for lower levels of regret found in previous studies could be men with lPCa focusing on the benefits of treatment: when weighing the assumed benefit (tumor removal) against the harm (impairment of erectile functioning), the side effects might tend to take a back seat [22]. This effect could be even more pronounced for self-paying patients.

### “[I have] decided too quickly” (RP, T3)

Time pressure is omnipresent in clinical practice. Nevertheless, this issue has not gained much attention in research yet. We showed that time pressure shortly after diagnosis predicted longer-term, but not short-term decisional regret. As mentioned above, side effects of treatment may be initially seen as a temporary problem that may still change. If there is no improvement in the long term, however, this could lead to regretting a decision having been made under time pressure. Another reason is that despite the good prognosis of lPCa, the word “cancer” is still often perceived as a “death sentence” [23]. This perception may increase anxiety and the desire for a rapid local approach. Defined time limits for treatment decision and initiation could amplify time pressure. However, for low/intermediate risk tumors, an oncological deterioration within three to six months after diagnosis is not very likely [24, 25]. Many men may not be aware of this. Experienced urologists therefore emphasize that men with lPCa have time to carefully weigh the pros and cons of each strategy. Nevertheless, individual patient factors (e.g. micturition at diagnosis, inconsistent diagnostic findings of biopsy and MRI) should be taken into account, potentially limiting this time frame. More precise diagnostic procedures (such as MRI-assisted biopsy) will provide patients with more certainty in future.

### “If I had known how this would affect me after surgery, I would have decided against therapy” (RP, T3)

Our finding, that erectile functioning predicts regret in the longer term, is consistent with other studies [9, 11, 13]. Speer et al. [23] showed that in the face of diagnostic shock, “hard outcomes” like survival and cancer eradication have the highest priority. Sexuality, as one aspect of quality of life, may often be of secondary importance in this phase, but regains importance over time. In addition, the relevance of sexuality is often overlooked because erectile dysfunction becomes more likely with age: in the general population more than 40% of men over 76 years report limitations in this regard [26]. However, since treatment groups did not differ at baseline, our findings show that a substantial proportion of the deterioration in erectile functioning is attributable to local treatment.

Satisfaction with sexual life, which captures psychological aspects of sexuality [27], is an important predictor for decisional regret. Sexual satisfaction may be more modifiable than erectile dysfunction and could thus be a starting point for interventions that aim at reducing regret. Our findings align with those of other studies [27, 28] highlighting the importance of satisfaction with sexual life as a patient-centered outcome for quality of life in cancer survivors. In future, patient-reported experience measures (PREMs), like satisfaction with sexual life, will become increasingly important. Interestingly, the satisfaction of men under AS decreased in the longer-term and approached the levels of men with RP/RT. The strain of living with a persistent cancer disease, affecting different areas of life, could add to this decrease.

### “Not enough information [was provided about] sparing methods” (RP, T2)

It has been well-documented that an informed decision can contribute to preventing decisional regret [e.g. 11]. In our study, however, the information by the urologist played a minor role. A possible reason for this may be a ceiling effect, since the large majority of men in our sample felt adequately informed by their urologist. Another explanation could be that the item we used rather reflects the trust in the urologist than the information itself. Objective measures (e.g. knowledge questions) could be helpful to assess how the amount and quality of medical information correspond to decisional regret. Furthermore, the grade of interdisciplinarity could depict the consultation quality. Finally, there are currently mixed findings on whether online tools can support informed decision-making and prevent regret [e.g. 29]. Further studies are needed to clarify these questions.

### Strengths and limitations

Strengths of the present study are one of the largest samples of AS patients with a longitudinal design and the low drop-out rate. Furthermore, we analyzed treatment received at every measuring point instead of using intention-to-treat analyses. This approach provides a realistic representation of different treatments and their respective side effects. There are also some limitations: (1) As this is an observational study, no causal conclusions can be drawn. However, since clinical experience shows that men have strong preferences for a particular treatment, it can be assumed that the high external validity of our study design outweighs the advantages of randomized assignment. (2) We cannot completely rule out the possibility of sampling effects, for example that men who are satisfied with their treatment could be more likely to participate. This could contribute to underestimating actual levels of decisional regret. (3) Since the sub-sample of men with RT is relatively small, the findings for this population should be interpreted with caution. However, the results offer valuable insights that can be used to generate hypotheses. Future studies should also be powered to differentiate different methods of RT. (4) We cannot generalize our results for men under focal therapies. A study by Westhoff and colleagues [30] identified a number of initial factors associated with regret in this patient population. Future studies should analyze the associations between regret, time pressure, and satisfaction with sexual life in men undergoing focal therapies. (5) We are aware that other treatment side-effects, such as urinary incontinence, are also predictive of decisional regret [9, 30]. However, since we focused on different aspects of sexuality, other side-effects were outside the scope of this paper. (6) We also did not assess why patients chose or changed to a particular treatment. This should be subject to future studies.

### Implications

The effects of time pressure have been neglected in cancer research so far. A first step would be the development of a standardized measurement instrument for assessing time pressure. Furthermore, standardized cut-off values for decisional regret are needed to facilitate interpretation of results.

Especially considering the good prognosis of lPCa and similar mortality rates between treatments [3], educating patients about side effects and sexuality in particular is key. However, the patient may have difficulties bringing up the subject of sexuality. Doctors may assume the patient has no need. The resulting collusion can be overcome through a routine of open questions. To alleviate time pressure, doctors can encourage patients to take enough time for treatment decision-making. In addition, a culture of shared decision making may contribute to reducing regret.

## Conclusions

Our study adds to the understanding of decisional regret in men with lPCa. While time pressure and impairment of erectile function increased the probability of regret in the longer-term, satisfaction with sexual life decreased the probability of regret in the short- and long-term. Enough time in the decision-making process and realistic expectations regarding treatment side effects may help to prevent regret.

## Supplementary Information

Below is the link to the electronic supplementary material.

Supplementary file1 (DOCX 14 kb)

Supplementary file2 (DOCX 17 kb)

Supplementary file3 (DOCX 14 kb)

Supplementary file4 (PPTX 51 kb)

## Data Availability

The datasets generated during and/or analyzed during the current study are not publicly available due participant anonymization but are available from the corresponding author on reasonable request.

## Abbreviations

AS: Active surveillance
E.g.: Exempli gratia
I.e.: Id est
lPCa: Localized prostate cancer
PCa: Prostate cancer
RP: Radical prostatectomy
RT: Radiation therapy

## Code availability

IBM SPSS Statistics—Version 25.

## References

[1] Bray F, Ferlay J, Soerjomataram I, Siegel RL, Torre LA, Jemal A (2018). Global cancer statistics 2018: GLOBOCAN estimates of incidence and mortality worldwide for 36 cancers in 185 countries. CA Cancer J Clin.

[2] Robert Koch-Institut (2019) Gesellschaft der Epidemiologischen Krebsregister In: Deutschland e.V. (eds) Krebs in Deutschland 2015/2016, 12th edn. Berlin

[3] Hamdy FC, Donovan JL, Lane JA, Mason M, Metcalfe C, Holding P (2016). 10-year outcomes after monitoring, surgery, or radiotherapy for localized prostate cancer. N Engl J Med.

[4] Donovan JL, Hamdy FC, Lane JA, Mason M, Metcalfe C, Walsh E (2016). Patient-reported outcomes after monitoring, surgery, or radiotherapy for prostate cancer. N Engl J Med.

[5] Chen RC, Basak R, Meyer A-M, Kuo T-M, Carpenter WR, Agans RP (2017). Association between choice of radical prostatectomy, external beam radiotherapy, brachytherapy, or active surveillance and patient-reported quality of life among men with localized prostate cancer. JAMA.

[6] Marzouk K, Assel M, Ehdaie B, Vickers A (2018). Long-term cancer specific anxiety in men undergoing active surveillance of prostate cancer: findings from a large prospective cohort. J Urol.

[7] Davison BJ, So AI, Goldenberg SL (2007). Quality of life, sexual function and decisional regret at 1 year after surgical treatment for localized prostate cancer. BJU Int.

[8] Lin Y-H (2011). Treatment decision regret and related factors following radical prostatectomy. Cancer Nurs.

[9] Wilding S, Downing A, Selby P, Cross W, Wright P, Watson EK (2020). Decision regret in men living with and beyond non-metastatic prostate cancer in the United Kingdom: a population-based patient-reported outcome study. Psycho-Oncol.

[10] Christie DRH, Sharpley CF, Bitsika V (2015). Why do patients regret their prostate cancer treatment? A systematic review of regret after treatment for localized prostate cancer. Psycho-Oncol.

[11] Hoffman RM, Lo M, Clark JA, Albertsen PC, Barry MJ, Goodman M, Penson DF, Stanford JL, Stroup AM, Hamilton AS (2017). Treatment decision regret among long-term survivors of localized prostate cancer: results from the prostate cancer outcomes study. J Clin Oncol.

[12] Feldman-Stewart D, Tong C, Brundage M, Bender J, Robinson J (2018). Prostate cancer patients’ experience and preferences for acquiring information early in their care. Can Urol Assoc J.

[13] Albkri A, Girier D, Mestre A, Costa P, Droupy S, Chevrot A (2018). Urinary incontinence, patient satisfaction, and decisional regret after prostate cancer treatment: a French national study. Urol Int.

[14] Tsiga E, Panagopoulou E, Sevdalis N, Montgomery A, Benos A (2013). The influence of time pressure on adherence to guidelines in primary care: an experimental study. BMJ Open.

[15] Inbar Y, Botti S, Hanko K (2011). Decision speed and choice regret: When haste feels like waste. J Exp Soc Psychol.

[16] Bergengren O, Garmo H, Bratt O, Holmberg L, Johansson E, Bill-Axelson A (2019). Determinants for choosing and adhering to active surveillance for localised prostate cancer: a nationwide population-based study. BMJ Open.

[17] Brehaut JC, O'Connor AM, Wood TJ, Hack TF, Siminoff L, Gordon E, Feldman-Stewart D (2003). Validation of a decision regret scale. Med Decis Making.

[18] Johansson E, Steineck G, Holmberg L, Johansson J-E, Nyberg T, Ruutu M, Bill-Axelson A (2011). Long-term quality-of-life outcomes after radical prostatectomy or watchful waiting: the Scandinavian Prostate Cancer Group-4 randomised trial. Lancet Oncol.

[19] van den Bergh RCN, Korfage IJ, Roobol MJ, Bangma CH, de Koning HJ, Steyerberg EW, Essink-Bot M-L (2012). Sexual function with localized prostate cancer: active surveillance vs radical therapy. BJU Int.

[20] van Stam M-A, Aaronson NK, Bosch JLHR, Kieffer JM, van Voort Zyp JRN, Tillier CN, Horenblas S, van der Poel HG (2020). Patient-reported outcomes following treatment of localised prostate cancer and their association with regret about treatment choices. Eur Urol Oncol.

[21] Rivera A, Scholar J (2020). Traditional masculinity: a review of toxicity rooted in social norms and gender socialization. ANS Adv Nurs Sci.

[22] Redelmeier DA, Dickinson VM (2011). Determining whether a patient is feeling better: pitfalls from the science of human perception. J Gen Intern Med.

[23] Speer SA, Tucker SR, McPhillips R, Peters S (2017). The clinical communication and information challenges associated with the psychosexual aspects of prostate cancer treatment. Soc Sci Med.

[24] Aas K, Fosså SD, Kvåle R, Møller B, Myklebust TÅ, Vlatkovic L, Müller S, Berge V (2019). Is time from diagnosis to radical prostatectomy associated with oncological outcomes?. World J Urol.

[25] Engl T, Mandel P, Hoeh B, Preisser F, Wenzel M, Humke C, Welte M, Köllermann J, Wild P, Deuker M, Kluth LA, Roos FC, Chun FKH, Becker A (2020). Impact of “time-from-biopsy-to-prostatectomy” on adverse oncological results in patients with intermediate and high-risk prostate cancer. Front Surg.

[26] Mazariego CG, Egger S, King MT, Juraskova I, Woo H, Berry M, Armstrong BK, Smith DP (2020). Fifteen year quality of life outcomes in men with localised prostate cancer: population based Australian prospective study. BMJ.

[27] Agochukwu NQ, Wittmann D, Boileau NR, Dunn RL, Montie JE, Kim T, Miller DC, Peabody J, Carlozzi NE (2019). Validity of the patient-reported outcome measurement information system (PROMIS) sexual interest and satisfaction measures in men following radical prostatectomy. J Clin Oncol.

[28] Wittmann D, Skolarus TA, Montie JE (2015). Are we targeting the right outcome for sexual health after prostate cancer treatment?. Eur Urol.

[29] Cuypers M, Lamers RED, Kil PJM, van de Poll-Franse LV, de Vries M (2019). Longitudinal regret and information satisfaction after deciding on treatment for localized prostate cancer with or without a decision aid. Results at one-year follow-up in the PCPCC trial. Patient Educ Couns.

[30] Westhoff N, Ernst R, Kowalewski KF, Schmidt L, Worst TS, Michel MS, von Hardenberg J (2020). Treatment decision satisfaction and regret after focal HIFU for localized prostate cancer. World J Urol.

