# Supplementary figures and images for: Time pressure predicts decisional regret in men with localized prostate cancer: data from a longitudinal multicenter study
Source: World J Urol. 2021 May 22;39(10):3755–61. doi: 10.1007/s00345-021-03727-0 (PMC8519821; doi:10.1007/s00345-021-03727-0)

## Slide 1
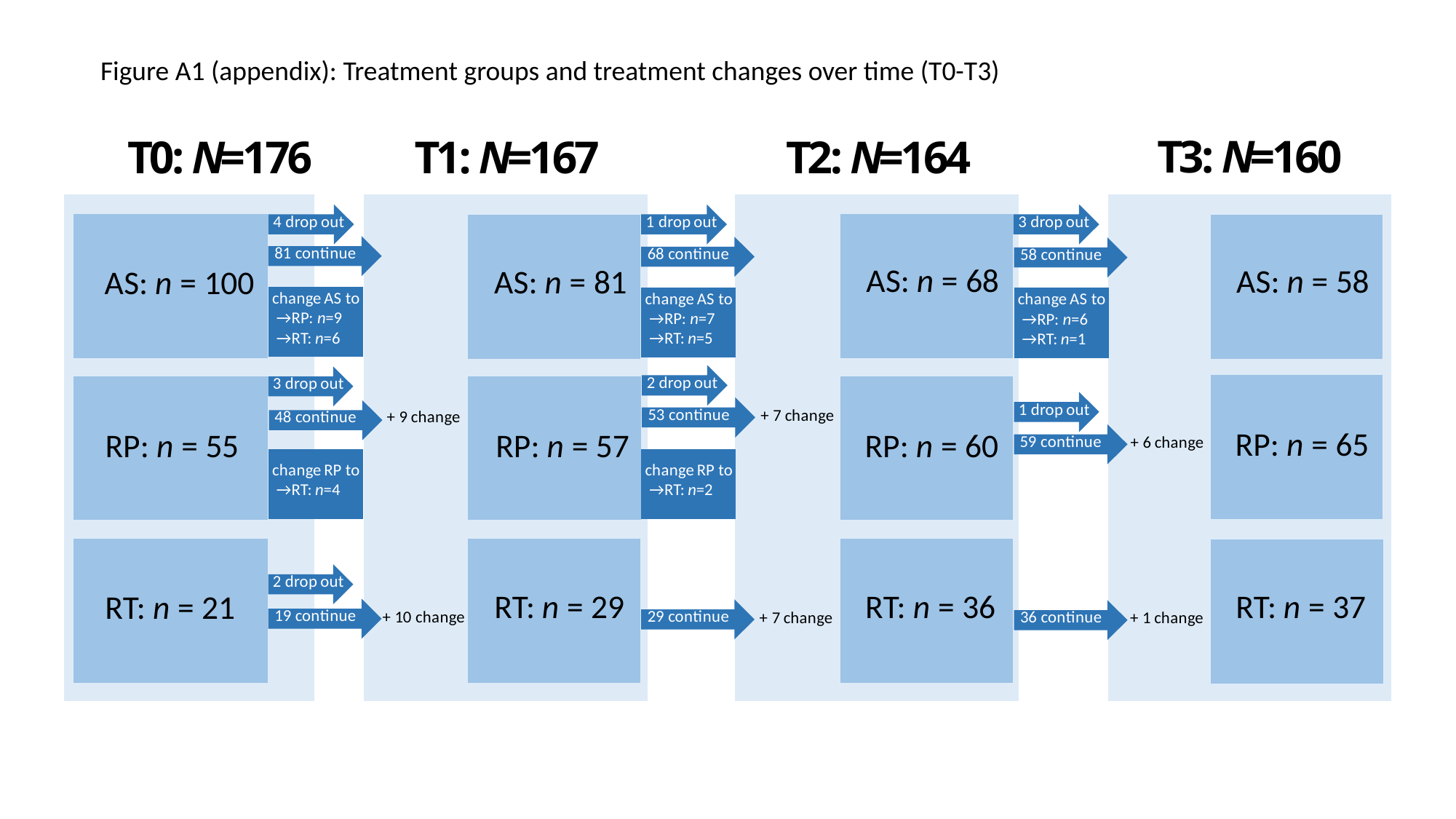

Figure A1 (appendix): Treatment groups and treatment changes over time (T0-T3)

Supplement: Supplementary file 1 — Supplementary file1 (DOCX 14 kb) [file 345_2021_3727_MOESM1_ESM.pptx]
