# Supplementary material for: Time pressure predicts decisional regret in men with localized prostate cancer: data from a longitudinal multicenter study
Source: World J Urol. 2021 May 22;39(10):3755–61. doi: 10.1007/s00345-021-03727-0 (PMC8519821; doi:10.1007/s00345-021-03727-0)
Supplement: Supplementary file 2 — Supplementary file2 (DOCX 17 kb) [file 345_2021_3727_MOESM2_ESM.docx]

**Table A2 (appendix)**: Medians and interquartile ranges/means and standard deviations of study variables according to treatment groups (sub-samples) and the respective effect sizes of group differences

|  | **Total** | **AS** | **RP** | **RT** | ***p*** | ***d****_RP-AS_* | ***d****_RP-RT_* | ***d****_RT-AS_* |
| --- | --- | --- | --- | --- | --- | --- | --- | --- |
| Impairment of erectile functioning, *Mdn (IQR)* |  |  |  |  |  |  |  |  |
| T0 | 2 (1) | 2 (1) | 2 (2) | 1 (1) | .35 | n.s. | n.s. | n.s. |
| T1 | 2 (2) | 2 (2) | 4 (1) | 2 (1) | <.001 | 1.7 | 1.1 | 0.4 |
| T2 | 3 (2) | 2 (2) | 3 (1) | 2 (2) | <.001 | 1.4 | 0.7 | 0.4 |
| T3 | 3 (2) | 2 (1) | 3 (1) | 2.5 (1) | <.001 | 1.1 | 0.8 | n.s. |
| Satisfaction with sexual life, *Mdn (IQR)* |  |  |  |  |  |  |  |  |
| T0 | 4 (1) | 3 (1) | 4 (2) | 4 (1) | .2 | n.s. | n.s. | n.s. |
| T1 | 3 (2) | 3 (2) | 2 (3) | 3 (2) | .002 | 0.6 | 0.6 | n.s. |
| T2 | 3 (2) | 4 (2) | 3 (2) | 4 (1) | .002 | 0.6 | 0.6 | n.s. |
| T3 | 3 (2) | 3 (2) | 3 (2) | 4 (2) | .11 | n.s. | n.s. | n.s. |
| Decisional regret, *M (SD)* |  |  |  |  |  |  |  |  |
| T1 | 12.5 (17.5) | 9.0 (12.2) | 15.2 (18.9) | 12.0 (20.6) | .28 | n.s. | n.s. | n.s. |
| T2 | 9.6 (14.4) | 5.6 (11.5) | 13.7 (17.2) | 8.2 (11.2) | .02 | 0.54 | n.s. | n.s. |
| T3 | 11.7 (14.0) | 8.3 (11.9) | 15.3 (15.7) | 10.6 (12.7) | .04 | 0.50 | n.s. | n.s. |

AS, active surveillance; RP, radical prostatectomy; RT, radiation therapy; *M*, arithmetic mean; *SD*, standard deviation; d, Cohen’s d, sample sizes: T0, *N*=176 (AS, *n*=100; RP, *n*=55; RT, *n*=21); T1, *N*=167 (AS, *n*=81; RP, *n*=57; RT, *n*=29); T2, *N*=164 (AS, *n*=68; RP, *n*=60; RT, *n*=36); T3, *N*=160 (AS, *n*=58; RP, *n*=65; RT, *n*=37); Time pressure, range: 1-4; Information provided by urologist, range: 1-4; Impairment of erectile functioning, range: 1-4; Satisfaction with sexual life, range: 1-5; Decisional regret, range: 0-100
