# Supplementary material for: Time pressure predicts decisional regret in men with localized prostate cancer: data from a longitudinal multicenter study
Source: World J Urol. 2021 May 22;39(10):3755–61. doi: 10.1007/s00345-021-03727-0 (PMC8519821; doi:10.1007/s00345-021-03727-0)
Supplement: Supplementary file 3 — Supplementary file3 (DOCX 14 kb) [file 345_2021_3727_MOESM3_ESM.docx]

**Table A3 (Appendix):** Frequencies and percentages of dichotomized study variables according to treatment groups (sub-samples)

|  | **Total** | **AS** | **RP** | **RT** |
| --- | --- | --- | --- | --- |
| Moderate/high time pressure (T0), *n (%)* | 48 (31.4) | 22 (25.6) | 21 (44.7) | 5 (25) |
| Sufficient information by urologist (T0), *n (%)* | 161 (91.5) | 90 (90) | 51 (92.7) | 20 (95.2) |
| Moderate/severe impairment of erectile functioning, *n (%)* |  |  |  |  |
| T0 | 39 (23.6) | 22 (22.7) | 14 (28.6) | 3 (15.8) |
| T1 | 78 (47.9) | 21 (26.3) | 48 (85.7) | 9 (33.3) |
| T2 | 83 (52.2) | 20 (30.8) | 49 (83.1) | 14 (40.0) |
| T3 | 96 (60.8) | 25 (43.1) | 53 (82.8) | 18 (50.0) |
| Moderate/high satisfaction with sexual life, *n (%)* |  |  |  |  |
| T0 | 137 (80.1) | 81 (81.8) | 37 (72.5) | 19 (90.5) |
| T1 | 104 (63.0) | 58 (71.6) | 26 (45.6) | 20 (74.1) |
| T2 | 104 (65.4) | 48 (72.7) | 30 (50.8) | 26 (76.5) |
| T3 | 96 (62.3) | 38 (66.7) | 33 (54.1) | 25 (69.4) |
| mild/severe decisional regret, *n (%)* |  |  |  |  |
| T1 | 51 (47.7) | 14 (41.2) | 26 (54.2) | 11 (44) |
| T2 | 52 (40.3) | 10 (23.8) | 29 (54.7) | 13 (38.2) |
| T3 | 62 (47.0) | 15 (31.9) | 32 (60.4) | 15 (46.9) |

AS, active surveillance; RP, radical prostatectomy; RT, radiation therapy; sample sizes: T0, *N*=176 (AS, *n*=100; RP, *n*=55; RT, *n*=21); T1, *N*=167 (AS, *n*=81; RP, *n*=57; RT, *n*=29); T2, *N*=164 (AS, *n*=68; RP, *n*=60; RT, *n*=36); T3, *N*=160 (AS, *n*=58; RP, *n*=65; RT, *n*=37)
