# Supplementary material for: Time pressure predicts decisional regret in men with localized prostate cancer: data from a longitudinal multicenter study
Source: World J Urol. 2021 May 22;39(10):3755–61. doi: 10.1007/s00345-021-03727-0 (PMC8519821; doi:10.1007/s00345-021-03727-0)
Supplement: Supplementary file 4 — Supplementary file4 (PPTX 51 kb) [file 345_2021_3727_MOESM4_ESM.docx]

**Table A1 (appendix)**: Odds ratios (OR), confidence intervals (CI) and *R²* according to Nagelkerke of the different logistic regression models

|  | **Decisional Regret (T1)** | | **Decisional Regret (T2)** | | **Decisional Regret (T3)** | |
| --- | --- | --- | --- | --- | --- | --- |
|  | OR (95% CI) | *R²* | OR (95% CI) | *R²* | OR (95% CI) | *R²* |
| Time pressure (T0) | 0.81 (0.35-1.87) | 0.149 | 2.10 (0.96-4.59)^✝^ | 0.150 | 2.28 (1.04-4.99)* | 0.124 |
| Information provided by urologist (T0) | 1.60 (0.31-8.40) | 0.119 | 1.97 (0.46-8.48) | 0.117 | 1.11 (0.30-4.06) | 0.089 |
| Impairment of erectile functioning (T1) | 1.51 (0.63-3.67) | 0.127 | 1.91 (0.83-4.37) | 0.127 | 1.43 (0.65-3.12) | 0.095 |
| Impairment of erectile functioning (T2) | - | - | 2.00 (0.87-4.61) | 0.133 | 2.32 (1.06-5.06)* | 0.138 |
| Impairment of erectile functioning (T3) | - | - | - | - | 3.40 (1.56-7.42)** | 0.174 |
| Satisfaction with sexual life (T1) | 0.18 (0.07-0.45)*** | 0.277 | 0.68 (0.31-1.47) | 0.116 | 0.39 (0.18-0.84)* | 0.142 |
| Satisfaction with sexual life (T2) | - | - | 0.44 (0.19-0.98)* | 0.150 | 0.44 (0.20-0.97)* | 0.119 |
| Satisfaction with sexual life (T3) | - | - | - | - | 0.44 (0.20-0.96)* | 0.104 |

OR, odds ratio; CI, confidence interval; *R², Nagelkerke’s R²*

^✝^ *p* <.10, * *p* <.05, ** *p* <.01, *** *p* <.001
